# A cross-sectional analysis identifies a low prevalence of *Plasmodium ovale* species **infections in symptomatic and asymptomatic individuals in Kilifi county, Kenya.

**DOI:** 10.12688/wellcomeopenres.17972.4

**Published:** 2024-09-17

**Authors:** Mercy Yvonne Akinyi, Margaret Chifwete, Leonard Ndwiga, Kelvin Muteru Kimenyi, Victor Osoti, Lynette Isabella Ochola-Oyier

**Affiliations:** 1 KEMRI-Wellcome Trust Research Programme, Kenya Medical Research Institute, Kilifi, P.O. Box 230, 80108, Kenya; 2 Institute of Primate Research, National Museums of Kenya, Nairobi, 24481-00502, Kenya; 3 Pwani University, Kilifi, P.O. Box 195-80108, Kenya; 4 Center for Biotechnology and Bioinformatics, University of Nairobi, Kilifi, P.O Box 30197-00100, Kenya

**Keywords:** Plasmodium ovale curtisi, malaria, PCR, Microscopy, Sequencing

## Abstract

**Background:**

The focus on *P. falciparum* diagnosis has led to an underestimation of the global burden of malaria resulting from neglected *Plasmodium* species. However, there is still scarce data on the prevalence of *P. ovale* species (spp) globally. To address this knowledge gap, data collected from cross-sectional studies in Kilifi county were used to: 1) determine the prevalence of *P. ovale spp* infections; and 2) determine the sensitivity of different diagnostic assays in detecting *P. ovale spp* infections.

**Methods:**

A total of 531 individuals were sampled across three study sites in Kilifi County, Kenya between 2009 and 2020. Blood smears were prepared from peripheral blood and screened for *Plasmodium* parasite stages using light microscopy. Molecular screening involved DNA extraction of dried blood spots and blood in ethylenediaminetetraacetic acid, polymerase chain reaction (PCR) using primers targeting the 18 small ribosomal subunit and sequencing.

**Results:**

Microscopy screening revealed that the most prevalent species was *P. falciparum* (32.0%) followed by *P. malariae* (9.0%) and then *P. ovale spp(* 1.5%). PCR screening identified additional *P. ovale spp* positives cases. Overall PCR results indicate that43 (8.1%) out of the 531 individuals harbored *P. ovale spp* infection with the highest prevalence reported in the tertiary health facility, (14.6%, 95% CI 8-23.6%), followed by the primary health facility (8.3%, 95% CI 5.4-11.9%), and the community from a cross-sectional blood survey, (3.6%, 95% CI 1.2-8.2%). Microscopy screening for *P. ovale spp* had a low sensitivity of 7% (95% CI 1-19-30%) and a high specificity of 99% (95% CI 98-100%). Sequencing results confirmed the presence of *P.ovale curtisi*.

**Conclusions:**

This study provides baseline data for *P.ovale spp* surveillance in Kilifi County, primarily using PCR to improve diagnosis. These results suggest that malaria elimination and eradication efforts should not only concentrate on *P. falciparum* but should embrace a holistic approach towards elimination of all *Plasmodium* spp.

## Introduction

Malaria is one of the leading causes of death especially in countries in Sub-Saharan Africa, South-East Asia and South America where it is highly endemic. In 2022, 249 million cases of malaria infection and 608,000 estimated deaths were reported globally with 94% of these cases occurring in Africa ^1^. The most affected populations being pregnant women and children under five years of age who are vulnerable to the disease. Despite significant decreases in malaria incidence and mortality among populations at risk in the recent past (2010–2019), there are still challenges to its elimination including antimalarial drug resistance, poorly understood asymptomatic infections, insecticide resistance, lack of sensitive diagnostic methods and poor access to affordable healthcare ^2, 3^.

The highest incidences of malaria related fatalities have been attributed to *Plasmodium falciparum* which is the most predominant species in Africa and accounted for 99.7% of malaria cases in 2019 ^3^. Additional *Plasmodium* parasites ( *P. vivax, P. ovale, P. malariae* and *P. knowlesi*) circulating in humans have also been associated with various forms of the disease and death. A recent review by Lover *et al*., (2018) ^4^ reports that most efforts of malaria eradication have focused on *P. falciparum,* whilst underestimating the burden of disease imposed by the remaining non-falciparum species infecting humans. For the successful elimination of malaria and monitoring of transmission patterns which are still unknown, knowledge of the epidemiology of neglected species is crucial ^5^.

*P. ovale* spp is one of the understudied *Plasmodium* species and mainly occurs in Sub-Saharan Africa and Asia ^6, 7^. It occurs in two morphologically identical but genetically distinct forms *P. ovale curtisi* and *P. ovale wallikeri* ^6^. Recent studies have shown that these *P.ovale* species provoke different clinical outcomes and duration of latency. *P. ovale wallikeri* is thought to be slightly more pathogenic than *P. ovale curtisi* because it has been associated with shorter duration of latency, and severe thrombocytopenia ^8, 9^. It is thus important to distinguish between these two *P.ovale* species. *P. ovale* spp infection can present as either benign tertian malaria or severe disease. Benign infections are characterized by low parasitemia, mild fever and occur for short durations while severe cases have been associated with jaundice, respiratory distress, thrombocytopenia and hypotension ^10, 11^. *P. ovale* spp has dormant hypnozoite liver stages that can cause relapse many months or years after initial exposure to sporozoites ^12^. 

Malaria infections by *P. ovale* spp rarely occur as mono-infections, instead, they co-occur with other *Plasmodium* species and hence they are easily missed during routine diagnosis. Due to its low parasitemia, detection of *P. ovale* spp using microscopy is extremely difficult and requires experienced technicians and good quality microscopes ^5^. In addition, most rapid diagnostic kits available in the market are specific to *P. falciparum* and hence less sensitive for detection of *P. ovale spp,* leading to false negative results ^13, 14^. Consequently, accurate diagnosis of *P. ovale spp* requires specific and sensitive methods such as nested polymerase chain reaction (PCR) tests or quantitative PCRs ^15, 16^. These tests are however expensive and not accessible to laboratories located in rural settings in malaria endemic regions. These diagnostic challenges further lead to the underestimation of the burden of malaria infection resulting from this neglected *Plasmodium* species that equally contributes to the global malaria burden.

In Kenya, few studies have investigated the prevalence of *Plasmodium ovale spp.* For instance, Tobian *et al*., (2000) ^16^ investigated the prevalence of malaria infection in Kwale county, in 102 paired maternal-blood and umbilical cord blood and found a prevalence of 24% for *P.ovale* spp via PCR. A second study conducted by Miller *et al*., (2015) ^17^ developed new qPCR assays for the detection of *P. ovale* spp in western Kenya. This new qPCR assays utilized primers designed from the reticulocyte binding protein (rbp2) gene which has conserved regions of both *P. ovale* spp that are absent in other falciparum and non-falciparum species. The authors reported better amplification using the rb2 gene target when compared to the qPCRs that are based on the 18 sRNA and tryptophan-rich antigen gene. They also reported a high specificity and ability to detect the parasite at low levels. However, this study did not determine the prevalence of *P.ovale* spp in the study area. Two studies conducted in Western Kenya also report similar low (0.8%) prevalences of *P. ovale* spp ^18, 19^. For close to three decades malaria surveillance efforts in Kilifi county have mainly focused on *P. falciparum* ^20, 21^. To our knowledge, there is a lack of data on the prevalence and trends of *P. ovale* spp in Kilifi county. To generate data for a proof-of-concept of molecular surveillance of *P. ovale* spp as an exemplar of non- *Pf* malarias, a convenient approach of archived samples, readily available primers and reagents were used to screen samples from cross-sectional studies in Kilifi county to: 1) determine the prevalence of *P. ovale spp* mixed and mono infections and 2) determine the sensitivity of different diagnostic assays in detecting *P. ovale* spp infections.

## Methods

### Study area and population

This study was conducted in Kilifi county in Kenya, which is a malaria endemic region characterized by long rains from April to July and short rains between November and December ^22^. The data used for this analysis were based on cross-sectional surveys from a primary healthcare facility, Pingilikani dispensary, where routine malaria monitoring is conducted, a community survey from a cohort of children living in Junju and a tertiary healthcare facility, Kilifi County Hospital (KCH) between 2009 and 2020. The above-mentioned populations reside in moderate to high malaria transmission areas as previously reported ^22^. Specifically, in the community survey, samples were collected from participants who were part of a cohort of children recruited at birth for weekly clinical surveillance until the age of 15. The samples were obtained from the annual cross-sectional blood survey from 2009–2019. The participants from the primary health facility were mainly children below 12 years of age who presented themselves for assessment at the dispensary from 2019–2020 as part of a malaria monitoring project at primary health care facilities. Samples from tertiary health facility were retrieved from the hospital surveillance population after testing positive for *P. falciparum* using rapid diagnostic test kits (RDTs) and microscopy from 2011–2018. Thin and thick blood smears were prepared from peripheral blood using standard protocols ^23^. This study was conducted in two phases. The first phase focused on 1) optimizing molecular conventional PCR screening for *P. ovale* spp and 2) screening community samples. During this phase of the study, we first selected non-pf microscopy samples that were available from the community (20 samples) from 2009–2015 to confirm the presence of *P. ovale* spp in the study area. Upon positive detection and confirmation of the *P. ovale* spp by conventional PCR screening and sequencing in the first set of samples, we then run all the available 119 community samples (Extended data ovale 4). The second phase of the study comprised of all available archived samples from the tertiary and primary health facilities regardless of their species microscopy positivity status (392 samples). The molecular screening involved conventional and/or RT-PCR screening and was dependent on available reagents at the time of screening (Extended data ovale 4. All positive RT-PCR samples underwent conventional PCR to enable sequencing. Summary of the sample distribution across the study sites can be found in the *Extended data* Table 1 ^24^.

### Ethics statement

The project was approved on 14 ^th^ November 2018 by the ethics review committee of the Kenya Medical Research Institute under protocol number SERU/CGMR-C/136/3753 and is renewed annually. Written informed consent in form of a written signature or a thumb print, including consent to utilization of samples and publication of research data, was obtained from all adult study participants and from legal guardians in cases where the participants were below 18 years old.

### DNA extraction and molecular detection of *Plasmodium ovale* by PCR

Total genomic DNA for blood samples from tertiary health facility and the community survey were extracted using QIAmp DNA Blood Mini kit, (Hilden, Germany) whereas for the primary health facility sample extraction was performed by using Qiacube HT robot, as per the manufacturers’ protocol.

PCR based screening for detection of *P. ovale* spp was done using conventional PCR assays and Real-time PCR. For the conventional assay, a nested PCR approach that targeted the 18 small ribosomal subunit (srRNA) of *P.* ovale spp was employed. The primary PCR reaction (N1) was done using *Plasmodium* genus specific primers rPLU5 and rPLU6 ^25^ and the Expand ^TM^ High Fidelity PCR system (Roche). The PCR was conducted using a Applied Biosystems VeritiTM Thermal Cycler and the conditions included initial denaturation at 94°C for 2 minutes, denaturation at 94°C for 30 seconds, annealing at 55°C for 30 seconds, elongation at 72°C for 1 minute and final extension for 7 minutes. The total number of cycles was 30, after which the products were allowed to cool at 20°C for 5 minutes. For the secondary reaction, 1µl of the PCR products from the first reaction was used as the template for secondary reaction (N2). The master mix prepared was similar with N1 except species specific primers were included to detect *P. ovale curtisi species* (r OVAL1 and r OVAL 2) ^26^. The PCR conditions were also similar to N1, except the annealing temperature was set at 58°C. PCR products (~800bp) were then visualised on a 1.5% gel stained with RedSafe ^TM^ (iNtRON). Available gel images provided as *Extended data* 2 ^24^. 

Real-time PCR assays for this study also targeted the small subunit of 18S rRNA of *P. ovale* spp, since it is a conserved region across *Plasmodium* parasites ^27^. This assay was performed using the Applied Biosystems™ 7500 Real-Time PCR System under universal conditions of 95°C for 15 minutes and 60°C for 1 minute with 45 cycles. The total reaction volume was 25µl containing 5µL of the template DNA, 12.5µl of Taqman universal master mix, 1µl of primers (for *P. ovale* spp the OVA-F forward primer Ova-F (50nm) and Plasmo2-R primer reverse primer (200nm) were used) and 0.25µl of probes (Ova probe (80nm). A cut-off of 40 cycles (i.e. CT Value of below 40) was used to define positive samples ^28^. Available raw RT_PCR data are shared as *Extended data* 3 ^24^. In addition, we retrieved *P. falciparum* PCR screening results for the tertiary healthcare facility to aid in identification of co-infections. These results were generated from a previous study that had just concluded.

### Capillary Sequencing and phylogenetic analysis

PCR products of *P. ovale* spp positive samples were purified using ExoSAP-IT (Thermo Fisher Scientific) and sequenced using BigDye Terminator v3.1 cycle sequencing kit v3.1 (Applied Biosystems, UK). An aliquot of 8.0µl of the BigDye master mix (0.5µl BigDye enzyme mix, 1.75µl BigDye sequencing buffer, 4.75µl nuclease free water and 1µl of 5µm r OVAL1 and r OVAL 2 primers) was added to 2µl of the purified template. The sequencing cycling conditions included; initial denaturation at 95°C for 30 seconds, 25 cycles of 96°C for 10 seconds, 50°C for 5 seconds, 60°C for 4 minutes; and a final hold of 20 minutes at 20°C. Capillary sequencing was done at the International Livestock Research Institute (ILRI, Kenya) using an ABI 3730xl capillary sequencer (Applied Biosystems).

The sequences obtained were assembled in CLC Main Workbench v7.9.1 (Qiagen, UK, RRID:SCR_000354) and consensus sequences extracted and compared to GenBank entries using BLASTN sequence homology searches ^29^. These sequences together with 18sRNA sequences from several *Plasmodium spp*. in PlasmoDB (Accession numbers PF3d7_0531600, PmUG01_10013000, PKNH_03209000, PVP01_0202900) and GenBank (Accession numbers KJ871672, KX672022, KF192073, MG847121 and KF21956) were then aligned using Multiple Sequence Comparison by Log Expectation (MUSCLE) ^30^ and used construct a neighbour-joining tree with 1,000 bootstrap replications ^31^ using SeaView version5.0.5 (RRID:SCR_015059) ^32^.

### Data analysis

All data analysis were performed using R statistical software version 3.6.3 (RRID:SCR_001905). The estimated prevalence of infection is presented at 95% confidence interval (CI). In addition, the epiR package in R was used to analyze the specificity and sensitivity of microscopy. We used the combined results of the conventional and RT-PCR as our gold standard. We also used Chi square tests (prop.test and prop.trends.test commands in R) to investigate if the proportion of positive *Plasmodium spp.* cases were similar across the study sites.

## Results

A total of 531 samples were screened for *Plasmodium spp* infection from three sites in Kilifi county: 139 from the community surveillance, 89 from the tertiary health facility and 303 from the primary health facility ( Table 1). Among the 531 samples, 50.1% (n =266) were female, 49.9% (n=265) were males and the median age of sampled individuals was 6.2 years (range ~5 months to 62.7 years). Overall, 528 individuals were below 15 years, whereas 3 individuals from primary health facility were aged 33, 55 and 62 years. 

**Table 1.** Prevalence of malaria species (mono and mixed infections) in the 3 study sites using microscopy.

Plasmodium infection status	Community (n=139)	Primary Health Facility (n=303)	Tertiary Health Facility (n=89)	Total (n=531)	% of each species calculated on the number of positive samples	P value from Chi square tests
No infection	88 (63.3%)	200 (66.0%)	0 (0.0%)	288 (54.2%)	-	
Pf only	27 (19.4%)	96 (31.7%)	47 (52.8%)	170 (32.0 %)	170 (70%)	<0.001
Pm only	17 (12.2%)	6 (2.0%)	25 (28.1%)	48 (9.0%)	48 (19.8%)	<0.001
Po only	6 (4.3%)	1 (0.3%)	1 (1.1%)	8 (1.5%)	8 (3.3%)	0.006
Pf-Pm	1 (0.7%)	0 (0.0%)	16 (17.9%)	17(3.2%)	17 (6.9%)	<0.001

No infection- absence of infection by any Plasmodium species, P.f- *Plasmodium falciparum*, P. o- *Plasmodium ovale spp*, P.m – *Plasmodium malariae*. Co-infections are denoted by hyphens. The percentages were calculated by number of cases (numerator) over the sample size (n) as the denominator.

### Prevalence of all Plasmodium species infection by microscopy

Microscopy results showed that 288 out of 531 samples (54.2%) did not harbor any *Plasmodium* parasites, whereas the rest had either mixed or mono infections by three *Plasmodium* species including *P. falciparum*, *P. ovale* spp and *P. malariae* ( Table 1). Across the three study sites, the most prevalent species was *P. falciparum* (32.0%, 95% CI 28.1-36.1%) followed by *P. malariae* (9.0%, 95% CI 6.7-11.8%) and the least prevalent species was *P. ovale* (1.5%, 95% CI 0.7-2.9%) ( Table 1). *P. falciparum* co-infection with *P. malariae* was also observed, particularly in patients from the tertiary health facility (3.2%, 95% CI 1.87-5.08%). The differences in prevalence of the *Plasmodium* species by site were significant (see Table 1).

### Sensitivity of microscopy and PCR diagnostic assays in detecting *P. ovale* spp mono and mixed infections

**Figure 1.**
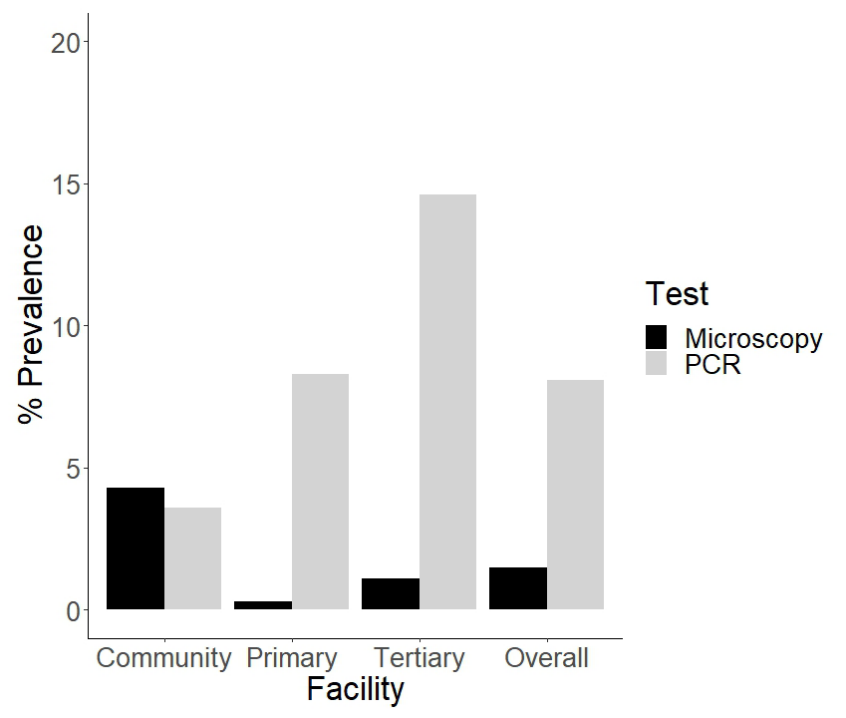
Barplot showing the percentage prevalence of *P. ovale* spp in the community, primary and tertiary facility. There is also an overall percentage prevalence that is calculated from the total prevalence. These percentages were calculated by number of positive cases (numerator) over the sample size (n) as the denominator.

Microscopy screening for *P. ovale* spp for across all samples had a low sensitivity of 7% (95% CI 1-19-30%) and a high specificity of 99% (95% CI 98-100%) ( Figure 1). Three out of the eight samples that tested positive by microscopy also tested positive by PCR for *P. ovale* spp, the rest were negative. Molecular screening for *P. ovale* spp (using conventional and real time PCR) revealed an increase in the prevalence of *P. ovale* spp infections when compared with microscopy. Overall, *P. ovale species* spp was detected by PCR in 43 out of the 531 samples (8.1%, 95 CI 5.9-10.8%). The highest prevalence was reported in the tertiary health facility (14.6%, 95% CI 8-23.6%), followed by the primary health facility (8.3%, 95% CI 5.4-11.9%) and then the community survey (3.6%, 95% CI 1.2-8.2%) ( Figure 1). The differences in *P.ovale* spp prevalence by site were significant. In total, 17 females and 26 males ranging from 5 months to 12 years harbored *P. ovale* spp infections.

PCR diagnostics also revealed additional mixed infections (when combined with *P.falciparum* and *P.malariae* microscopy results) in the community survey, 40% (2) of the PCR positive *P. ovale* spp infections were mono infections, 40% (2) mixed infection with *P. falciparum* and 20% (1) with *P.malariae*. The tertiary health facility only had one individual with *P. ovale* spp mono infection (7.7%(1)), the rest of the cases presented as triple infections (53.8% (7)) and dual infections with *P. malariae* (38.5% (5)).

Fortunately, the primary health facility data set had microscopy results for all *Plasmodium* species and PCR results for both *P. falciparum* and *P.ovale* spp, thus increasing our ability to detect *falciparum-ovale* mixed infections. Overall, 24% (6) of the *P. ovale* spp PCR positive cases were *P. ovale* spp mono infections, 68% (17) mixed infections with *P. falciparum*, 4% (1).

mixed infections with *P.malariae* and 4% (1) mixed infections with all the three species identified in this study. We provide a summary of screening tests done, the sample sizes as well as results in extended data 4.

### Sequencing results

**Figure 2.**
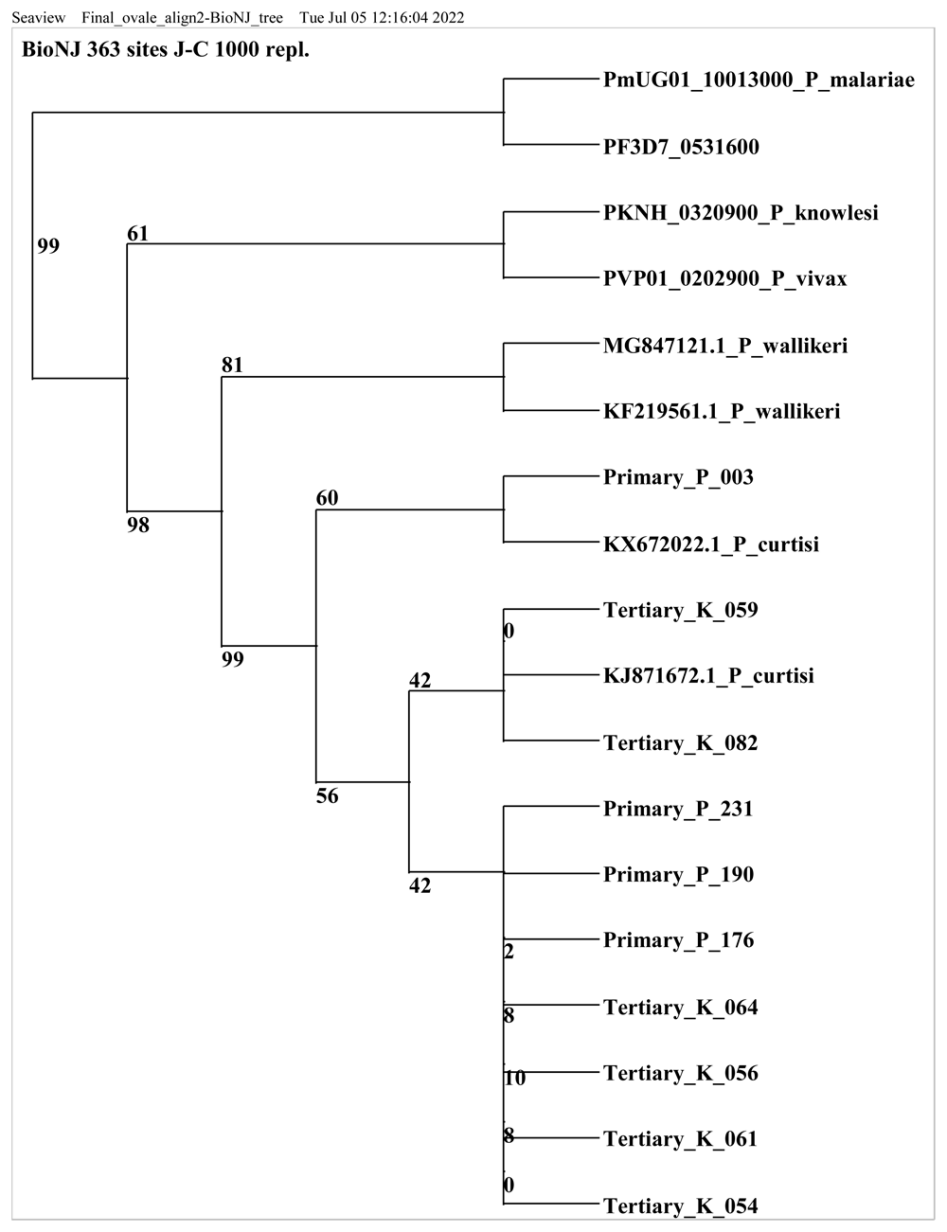
Phylogenetic tree of *Plasmodium ovale curtisi* positive samples based on18sRNA gene. Sequences obtained from GenBank and PlasmoDB are indicated by their accession numbers. Percentage bootstrap supports (1000 replicates) are shown by numbers at the respective nodes. *P. ovale* spp sequences retrieved from this study include those with Primary and tertiary prefixes.

All the 43 *P.ovale* spp positive samples underwent conventional PCR screening and samples with very faint or no bands (n=30) during gel electrophoresis were excluded from downstream processing for sequencing. Thirteen samples were thus selected for sequencing based on the band intensity of the conventional PCR amplicon. Ten of the 13 positive isolates were successfully sequenced and a nucleotide blast similarity search on GenBank confirmed the presence of *P. ovale curtisi* species in Kilifi county. Three sequences were excluded because they did not meet the set sequence quality threshold. Sequences retrieved from this study showed close homology (99% to 100% identity) with West African and China-Mynamar *P. ovale curtisi* isolates, accession numbers KJ871672, KX672022 and KF192073 (Li *et al*., 2014 ^33^; Li *et al*., 2016 ^34^, Figure 2). All sequences generated from this study were deposited in GenBank accession numbers MZ927719- MZ927728.

## Discussion

In Africa, surveillance of non-falciparum malaria species has mostly been conducted in Western and Central Africa. Few studies have been conducted in Eastern and Southern Africa where malaria is also known to be endemic. This is the first study to investigate the prevalence of *P. ovale* spp in children in Kilifi county using both microscopy and PCR diagnostic methods, revealing a low prevalence of *P.ovale spp* (8.1%) in a relatively small sample set of individuals across three convenient sampling sites. Overall, we obtained similar results to those from microscopic surveillance studies in various African countries including Senegal and Ghana ^35– 37^. These studies reported a low prevalence of *P.ovale* spp with the highest burden in children below 10 years. In western Kenya, microscopic surveillance of *P. ovale* spp equally reported low prevalences of *P. ovale* spp, 0.79% in Rusinga Island ^18^ 0.8% in the Western Kenya highlands ^19^. Molecular surveillance using *P.ovale curtisi* specific primers revealed a low prevalence of the parasite in various studies; 8.3% in Democratic Republic of Congo ^38^, 2.1% in Zambia ^39^, 4.7 in Tanzania ^5^, 14.8% in Nigeria ^40^, and 7% in Uganda ^41^.

The low prevalence of *P.ovale* spp in this study can be attributed to the low parasite densities and short durations of patent infection that do not meet the parasite detection threshold ^7^. Low parasitemia (often below 1%) in *P. ovale* spp infected individuals is not uncommon because these parasites only infect young erythrocytes (reticulocytes) ^42^. Secondly, they are potentially sensitive to current antimalarial drugs during the treatment or chemoprophylaxis of *P. falciparum.* For example a study in Gabon reported efficacy of artemisinin-based combination therapies against *P.ovale* spp ^43^. However, Dinko *et al*., (2013) ^44^ and Betson *et al*., (2014) ^45^ reported persistent infection with both species of *P. ovale* post artemether-lumefantrine treatment. Further studies should investigate responses of non-falciparum species to antimalarial drugs. Thirdly, the intensification of malaria control strategies in most endemic areas, such as the use of insecticide treated nets, could also contribute to the low prevalence of non-falciparum species because the target mosquitoes are known to transmit all *Plasmodium* species ^46^. The study sample size is a possible limitation, since it was small and mostly focused on children below 15 years. Future endeavors should focus on screening a wider age group because previous studies have reported *P. ovale* spp infections in adults over 40 years old ^36^.

In this moderate-high transmission area, it was anticipated that patients presenting at the tertiary heath facility and the primary health facility would have a higher prevalence of *P. ovale* spp, because the infections were symptomatic. There was thus an under-estimation of *P. ovale* spp in symptomatic children seeking treatment and an under-estimation of the potential burden of disease associated with *P. ovale* spp. Participants from the community cross-sectional survey were mostly asymptomatic children and were likely to act as reservoirs for the malaria parasite. The lack of observed symptoms accompanied by lack of testing may facilitate transmission of *P. ovale* spp. Contrary to our findings, Faye *et al*., (2002) ^47^ reported a higher prevalence of *P. ovale* spp infections via microscopy screening in symptomatic children compared to asymptomatic ones in a high malaria transmission region in Senegal.

It is evident that there is an underestimation of *P. ovale* spp prevalence in malaria endemic countries because overall microscopy identified about five-fold fewer infections compared to PCR of the highly conserved 18sRNA gene. The low sensitivity of microscopic diagnosis can be attributed to low parasite densities or lack of skilled microscopists with sufficient experience to morphologically differentiate *P.ovale* spp from other malaria species. The continued use of microscopy as a gold standard for diagnosis of *Plasmodium* species will lead to the underdiagnosis of an already rare non-falciparum infection. Microscopic analysis was therefore complemented with PCR assays, since even the differentiation between *P. ovale curtisi* and *P. ovale wallikeri* are normally identified at the molecular level. Molecular diagnostic assays are more specific and sensitive especially for parasites that present with low densities such as *P. ovale curtisi* infections. However, as expected, due to the technical challenges in running PCRs and the costly acquisition and maintenance of equipment, these assays are not feasible in the clinical setting. This preliminary survey focused on determining whether *P.ovale* spp was circulating in the Kilifi population and capillary sequencing confirmed the presence of *P. ovale curtisi* species. The differences in prevalence of *P. ovale* spp based on microscopy and molecular screening especially in the community survey in this study could possibly be explained by the fact that samples from this population were only screened using primers specific for *P.ovale curtisi*. During this preliminary study we lacked a true positive control to optimize the *P.wallikeri* primers we had and therefore could not test for this *P.ovale* species. A follow-up study with a bigger sample size will be conducted to determine the prevalence of both *P. ovale* spp using conventional primers that simultaneously detect both species ^48– 52^.

Despite identifying 43 *P.ovale* spp positive samples, only 13 were selected for sequencing probably due to low parasitemia levels. The samples selected for sequencing were amplified using primers that were specific for *P. ovale curtisi* hence the lack of *P.ovale wallikeri* identification during sequencing. It is however possible that the RT-PCR primers picked both species of *P. ovale* and Phylogenetic analysis showed that the sequences retrieved from this study was within a well-supported *P. ovale* spp. Clade. The close homology (99% to 100% identity) of our sequences with the West African and China-Mynamar *P. ovale curtisi* isolates confirms that the 18S rRNA locus is highly conserved in this species and hence is an accurate diagnostic tool that should continue to be used for detection and identification of this species globally. The low parasitemia levels associated with non-falciparum species present challenges for studies that focus on molecular identification and characterization of circulating parasites and screening for other molecular markers, such as those for drug resistance.

Consistent with previous non-falciparum surveillance studies, this study reported both single and mixed infections of *P. ovale* spp with either *P. falciparum* or *P. malariae* or with triple infections by all species. Host responses to infection by one *Plasmodium* species may alter responses to infection by additional parasite species. For instance, individuals that have existing *P. falciparum* infection experience severe disease when co-infected with *P. vivax*, yet individuals who have existing *P. vivax* infection experience reduced severity to disease when co-infected with *P. falciparum* ^53^. Immune responses to parasite co-infections are complex and are dependent on the parasite in question and the order of infection. Studies on parasite interactions between *P. ovale* spp and other *Plasmodium* species in this study area are yet be conducted.

## Conclusions

This study provides baseline data for *P. ovale* spp molecular surveillance studies in Kilifi County. We report a low prevalence of *P.ovale* (8.1%) in a small sample set of 531 individuals across three study sites. *P.ovale* spp occurred as both mono and mixed infections with other *Plasmodium* species. We also show that there are still challenges in diagnosis of *P. ovale* spp because most settings still rely heavily on microscopy which has low sensitivity and rapid diagnostic kits (RDTs) which primarily identify *P. falicparum* and panRDTs which are not species specific. *Plasmodium* mixed infections can alter disease severity and morbidity, therefore clinical laboratories should use accurate combinations of diagnostic procedures or repeat blood film examinations by microscopists to identify co-infections in suspected patients ^54, 55^. This is crucial for therapeutic decisions, prompt treatment, and effective management among those patients ^54, 55^. The presence of *P. ovale* spp in this region suggests that malaria elimination and eradication efforts should not only concentrate on *P. falciparum but* should embrace a holistic approach towards elimination of all *Plasmodium* species. Future *P. ovale* spp surveillance projects should focus on determination of species prevalence, drug resistance markers, and risk factors associated with *P. ovale* spp transmission in this region. In addition, there is a need to innovate and adopt more sensitive diagnostic approaches to identify non-falciparum malaria.

## Data Availability

NCBI Nucleotide: All sequences generated from this study were deposited in GenBank. Accession numbers MZ927719 to MZ927728. Harvard Dataverse: Data for: A cross-sectional analysis identifies a low prevalence of Plasmodium ovale curtisi infections in symptomatic and asymptomatic individuals in Kilifi county, Kenya. https://doi.org/10.7910/DVN/AGTFDH ^24^. This project contains the following underlying data: Akinyi *et al*. Ovale_27Jun22_de-identified.xlsx. (This is the main data set for the Akinyi *et al*. *Plasmodium ovale* manuscript. This data set provides information on the prevalence of *Plasmodium ovale* across 3 sites in Kilifi county Kenya and demographic information of study participants. It also provides microscopy and/or PCR data for *Plasmodium falciparum* and *Plasmodium malariae*.) Data Readme File_OvaleMS.txt. (This file provides a summary of the study including the general project overview, ethical approval information, available datasets, terms of use, data content, method and processing.) KWTRP_DATA_CODEBOOK_OvaleMS.docx. (This file provides a summary of all data sets associated with the study and a description of their contents.) Harvard Dataverse: Data for: A cross-sectional analysis identifies a low prevalence of Plasmodium ovale curtisi infections in symptomatic and asymptomatic individuals in Kilifi county, Kenya. https://doi.org/10.7910/DVN/AGTFDH ^24^. This project contains the following extended data: Akinyi *et al*. Extended data_Ovale.xlsx. (This file contains supplemental data on the Akinyi *et al*. *Plasmodium ovale* manuscript. It details the sample distribution across the 3 study sites from 2009–2020). Akinyi *et al*. Extended data_Ovale_2.pdf. (This file contains supplemental data on the Akinyi *et al*. *Plasmodium ovale* manuscript. It contains gel images for a subset of the samples post conventional PCR screening.) Akinyi *et al*. Extended_data_Ovale_3.docx. (This file contains supplemental data on the Akinyi *et al*. *Plasmodium ovale* manuscript. It contains a flowchart describing sample numbers and screening tests conducted and PCR results table. Data are available under the terms of the Creative Commons Attribution 4.0 International license (CC-BY 4.0).
