# Supplementary material for: A cross-sectional analysis identifies a low prevalence of Plasmodium ovale species  infections in symptomatic and asymptomatic individuals in Kilifi county, Kenya
Source: Wellcome Open Res. 2024 Sep 17;7:207. Originally published 2022 Aug 11. [Version 4] doi: 10.12688/wellcomeopenres.17972.4 (PMC11447441; doi:10.12688/wellcomeopenres.17972.4)
Supplement: Peer review history

# Reviewer report: Reviewer response for version 4

**DOI:** 10.21956/wellcomeopenres.25427.r99758

**Published:** 2024-10-02

**Authors:** Valentin Joste

**Affiliations:** 1 National Malaria Reference Centre, AP-HP, Hôpital Bichat - Claude Bernard, University of Paris, Paris, France

The authors have answered all my questions and I have no further queries.

Is the work clearly and accurately presented and does it cite the current literature?

Yes

If applicable, is the statistical analysis and its interpretation appropriate?

Partly

Are all the source data underlying the results available to ensure full reproducibility?

Yes

Is the study design appropriate and is the work technically sound?

Partly

Are the conclusions drawn adequately supported by the results?

Yes

Are sufficient details of methods and analysis provided to allow replication by others?

Partly

Reviewer Expertise:

Plasmodium diagnosis; molecular biology

I confirm that I have read this submission and believe that I have an appropriate level of expertise to confirm that it is of an acceptable scientific standard.

# Reviewer report: Reviewer response for version 4

**DOI:** 10.21956/wellcomeopenres.25427.r99759

**Published:** 2024-09-18

**Authors:** Benoit Malleret

**Affiliations:** 1 Department of Microbiology and Immunology, Immunology Translational Research Programme, Yong Loo Lin School of Medicine, National University of Singapore, Singapore, Singapore

I have no further comments.

Is the work clearly and accurately presented and does it cite the current literature?

Yes

If applicable, is the statistical analysis and its interpretation appropriate?

Not applicable

Are all the source data underlying the results available to ensure full reproducibility?

Yes

Is the study design appropriate and is the work technically sound?

Yes

Are the conclusions drawn adequately supported by the results?

Yes

Are sufficient details of methods and analysis provided to allow replication by others?

Yes

Reviewer Expertise:

Malaria,  Parasitology, Innate Immunity, Microbiome

I confirm that I have read this submission and believe that I have an appropriate level of expertise to confirm that it is of an acceptable scientific standard.

# Reviewer report: Reviewer response for version 3

**DOI:** 10.21956/wellcomeopenres.22846.r70993

**Published:** 2024-01-31

**Authors:** Valentin Joste

**Affiliations:** 1 National Malaria Reference Centre, AP-HP, Hôpital Bichat - Claude Bernard, University of Paris, Paris, France

**General:**

 The changes made have considerably improved the manuscript. I have a few more general comments to make:

- Instead of *P. ovale* species or *P. ovale*, write *P. ovale* spp.
- Please correct subspecies with species throughout the manuscript.
- Please add line numbers for convenience

**Abstract**:

- « Overall PCR results … *P. ovale curtisi* infection » : This sentence refers to the combined results of conventional PCR and qPCR. As you were unable to differentiate the two species by qPCR, please replace *P. ovale curtisi* with *P. ovale* spp. in this sentence.

**Introduction**:

- Please indicate the number of cases and deaths in the latest WHO malaria report: https://www.who.int/teams/global-malaria-programme/reports/world-malaria-report-2022

**Results**:

- Number of conventional PCR performed: 378 in the general table in supplemental and 349 (210+139) in the flow-chart. Please check your numbers and correct them if necessary.
- For the 349 isolates subjected to conventional screening, only *P. ovale curtisi* was tested. Why was this? This probably explains the discrepancies between *P. ovale* detected by microscopy and not by PCR (J_002, J_003, J_007, J_024, P_222). It might be interesting to discuss these discrepancies in the discussion.
- You mention « The differences in *P. ovale* prevalence by sites were significant » but not statistical result is presented.
- From « PCR diagnostics also revealed… » to « identified in this study »: please add the number in () next to the percentages.
- For Sanger sequencing, the samples were selected from the total PCR *P. ovale *positive samples or from the conventional PCR *P. ovale* positive samples? How do you determine the band intensity for the qPCR *P. ovale* positive sample? Please consider clarify this section.
- Phylogenetic tree: does the 99 boostrap value belong to the *P.malariae* + *P.falciparum* clade or to the big clade below?

**Discussion**:

- Please discuss the fact that you did not detect *P. ovale wallikeri* in conventional PCR
- « Low parasitemia (often below 5%) » please change 5% with 1%

Is the work clearly and accurately presented and does it cite the current literature?

Yes

If applicable, is the statistical analysis and its interpretation appropriate?

Partly

Are all the source data underlying the results available to ensure full reproducibility?

Yes

Is the study design appropriate and is the work technically sound?

Partly

Are the conclusions drawn adequately supported by the results?

Yes

Are sufficient details of methods and analysis provided to allow replication by others?

Partly

Reviewer Expertise:

Plasmodium diagnosis; molecular biology

I confirm that I have read this submission and believe that I have an appropriate level of expertise to confirm that it is of an acceptable scientific standard, however I have significant reservations, as outlined above.

# Response

**Published:** 2024-09-06

**Authors:** Mercy Akinyi

**Affiliations:** KEMRI Wellcome Trust, Kenya

**We thank the reviewer for taking time to go through our revised manuscript. We address the reviews raised below in bold text.**

**General:** The changes made have considerably improved the manuscript. I have a few more general comments to make:

- Instead of *P. ovale *species or *P. ovale*, write *P. ovale* spp
**Response: We have now made these corrections as proposed.**
- Please correct subspecies with species throughout the manuscript
**Response: We have now made these corrections as proposed.**
- Please add line numbers for convenience.
**Response: We agree that this would help with the review process. However, the journal gave as a specific format that we have adhered to. **  
**Abstract: ** Overall PCR results … *P. ovale* infection: This sentence refers to the combined results of conventional PCR and qPCR. As you were unable to differentiate the two species by qPCR, please replace *P. ovale* curtisi with *P. ovale *spp.. in this sentence.
**Response: We thank the reviewer for pointing this out. We have corrected the sentence to read P. ovale spp.**  
**Introduction:** Please indicate the number of cases and deaths in the latest WHO report https://www.who.int/teams/global-malaria-programme/reports/world-malaria-report-2022
**Response: We have provided statistics from the latest world malaria 2023 report. “In 2022, 249 million cases of malaria infection and 608,000 estimated deaths were reported globally with 94% of these cases occurring in Africa.”**  
**Results:** Number of conventional PCR performed: 378 in the general table in supplemental and 349 in the flowchart. Please check your numbers cand correct them if necessary.
**Response: Thank you pointing us to the flowchart, now referred to as extended data _ovale_3 to confirm the sample numbers.  The flow chart indicates that samples screened by conventional PCR only were 349. In addition to these 349, RT-PCR positive samples were also screened by conventional PCR to enable downstream sequencing. The total number of samples that underwent conventional PCR screening therefore are 378 (349+16+13) which is in agreement with the main data set.  **
***         ***
  For the 349 isolates subjected to conventional screening, only *P. ovale curtisi* was tested, why was this?
**Response: As previously mentioned in the manuscript, and the responses to the reviewers, we were very eager to test the samples for *P.ovale wallikeri* as well but were unable to secure a reliable positive control for the same when the study was conducted. **
  This probably explains the discrepancies between *P. ovale* detected by microscopy and not by PCR (J_002, J_003, J_007, J_024, P_222). It might be interesting to discuss these discrepancies in the discussion.  
**Response: We welcome this suggestion by the reviewer and have now added this aspect in the discussion as a limitation of the study. “The differences in prevalence of *P. ovale* spp based on microscopy and molecular screening especially in the community survey in this study could possibly be explained by the fact that samples from this population were only screened using primers specific for *P.ovale curtisi.* During this preliminary study we lacked a true positive control to optimize the *P.wallikeri *primers we had and therefore could not test for this *P.ovale* species.”**  
  You mention the differences in P.ovale prevalence by sites were significant but not statistical result is presented.
**Response: The clarification on the statistical tests conducted was indicated in the data analysis section “We also used Chi square tests (prop.test and prop.trends.test commands in R) to investigate if the proportion of positive *Plasmodium spp.* cases were similar across the study sites.” In addition, P values of the tests were added in the last column of table 1. **
  From to identified in this study> please add the number in () next to the percentages             
**Response:** **The numbers have now been included as proposed**.
  For sanger sequencing, the samples were selected from the total PCR P.ovale positive samples or from the conventional PCR P.ovale positive samples. How do you determine the band intensity for the q PCR P. ovale spositve sample? Please consider clarify this section.
**Response: We have revised the text in this section. All qPCR positive samples underwent conventional PCR screening. The revised text reads as follows “All 43 *P.ovale *spp positive samples underwent conventional PCR screening and samples with very faint or no bands (n=30) during gel electrophoresis were excluded from downstream processing for sequencing. Thirteen samples were thus selected for sequencing based on the band intensity of the conventional PCR amplicon.”**
  Phylogenetic tree: does the 99 bootstrap value belong to the p.malariae +P. falciparum clade or to the big clade below?
**Response:** **The 99 bootstrap value belongs to the P .malariae + P. falciparum clade**  
**Discussion** Please discuss the fact that you did not detect P.ovale wallikeri in conventional PCR.
**Response: We clarified our inability to detect *P. ovale wallikeri* in the discussion section of the last version of the manuscript and also in the response to reviewers. We have also included a few sentences to emphasize on this limitation. “The differences in prevalence of *P. ovale* spp based on microscopy and molecular screening especially in the community survey in this study could possibly be explained by the fact that samples from this population were only screened using primers specific for *P.ovale curtisi.* During this preliminary study we lacked a true positive control to optimize the *P.wallikeri *primers we had and therefore could not test for this *P .ovale* species.”**
  Please change 5% with 1%
**Response: We have changed this to 1%.**       

# Reviewer report: Reviewer response for version 3

**DOI:** 10.21956/wellcomeopenres.22846.r70994

**Published:** 2023-12-16

**Authors:** Benoit Malleret

**Affiliations:** 1 Department of Microbiology and Immunology, Immunology Translational Research Programme, Yong Loo Lin School of Medicine, National University of Singapore, Singapore, Singapore

No further comments.

Is the work clearly and accurately presented and does it cite the current literature?

Yes

If applicable, is the statistical analysis and its interpretation appropriate?

Not applicable

Are all the source data underlying the results available to ensure full reproducibility?

Yes

Is the study design appropriate and is the work technically sound?

Yes

Are the conclusions drawn adequately supported by the results?

Yes

Are sufficient details of methods and analysis provided to allow replication by others?

Yes

Reviewer Expertise:

Malaria,  Parasitology, Innate Immunity, Microbiome

I confirm that I have read this submission and believe that I have an appropriate level of expertise to confirm that it is of an acceptable scientific standard.

# Reviewer report: Reviewer response for version 2

**DOI:** 10.21956/wellcomeopenres.22212.r67417

**Published:** 2023-09-29

**Authors:** Valentin Joste

**Affiliations:** 1 National Malaria Reference Centre, AP-HP, Hôpital Bichat - Claude Bernard, University of Paris, Paris, France

This work is of great interest and brings really interesting data to the scientific community. The text is easier to understand, and I thank the authors for this effort. But there are still problems with the number of *P. ovale* spp positive samples and I haven't been able to come up with the same results as those given by the authors in the text. Above all, I don't understand how the authors differentiated between *P. ovale curtisi *and *P. ovale wallikeri *when it seems to me that the qPCR they use detects both species. For example, in the sentence "Overall, *P. ovale curtisi *was detected by PCR in 48 of 531 samples (9.0%, 95 CI 6.7-11.8%)", I assumed that these results came from conventional PCR and qPCR?

 General remark : *P. ovale curtisi* and *P. ovale wallikeri* are considered two distinct species and not subspecies (see articles written by Colin Sutherland for more information on this subject). Please change subspecies to species in the entire document.

 Introduction:

- you cite the work of Miller *et al* but do not give the prevalence of P. ovale spp they found with their newly developed qPCR. This is interesting information.

Results: 

- you said you used a Ct threshold of 40 for qPCR results, but in the extended Table 3, you gave us positive qPCR results with Ct > 40 ;
- you wrote: "Three of the eight samples that tested positive by microscopy also tested positive by PCR" -> the remaining five samples were PCR negative? Also for other Plasmodium species?
- In the extended table 3, you had 21 isolates with a Ct < 40 for *P. ovale*. But in the general table with the combined results of qPCR and conventional PCR, you have 31 isolates with a Ct < 40. How do you explain this difference between the two tables? 
- You say you detected *P. ovale curtisi *in 48 isolates, but when I looked at the results in the overall table, only 43 isolates had a yes in the *P.ovale*_PCR overall column. Furthermore, as only 21 isolates were positive in conventional PCR, how can you differentiate *P. ovale curtisi* from *P. ovale wallikeri* with qPCR results alone?

I'm convinced that by clearly explaining in the text the number of positives by conventional PCR and/or qPCR, and by correcting the numbers of positives to be consistent with the extended table, the article will gain in comprehension.

Is the work clearly and accurately presented and does it cite the current literature?

Yes

If applicable, is the statistical analysis and its interpretation appropriate?

Partly

Are all the source data underlying the results available to ensure full reproducibility?

Yes

Is the study design appropriate and is the work technically sound?

Partly

Are the conclusions drawn adequately supported by the results?

Yes

Are sufficient details of methods and analysis provided to allow replication by others?

Partly

Reviewer Expertise:

Plasmodium diagnosis; molecular biology

I confirm that I have read this submission and believe that I have an appropriate level of expertise to confirm that it is of an acceptable scientific standard, however I have significant reservations, as outlined above.

# Response

**Published:** 2023-11-30

**Authors:** Mercy Akinyi

**Affiliations:** KEMRI Wellcome Trust, Kenya

**P. ovale reviewer comments ** This work is of great interest and brings really interesting data to the scientific community. The text is easier to understand, and I thank the authors for this effort. But there are still problems with the number of *P. ovale* spp positive samples and I haven’t been able to come up with the same results as those given by the authors in the text. **We thank the reviewer for taking time to go through our revised manuscript. We address the reviews raised below in bold text.**

 Above all I don’t understand how the authors differentiated between *P. ovale curtisi* and *P. ovale wallikeri* when it seems to me that the qPCR they use detects both species. For example in the sentence “overall, *P. ovale curtisi* was detected by PCR in 48 of 531 samples (9.0%, 95CI 6.7-11.8%”, I assumed that these results came from conventional PCR and qPCR? **The reviewer is correct about our inability to differentiate between the two subspecies using qPCR. We have now corrected the section to read “ *P. ovale species” *and gone through the entire manuscript to ensure consistency on the species term *.* Thank you for the correction on the prevalence of *P. ovale* using PCR, we have now revised this to read .. “Overall PCR results indicate that 43 (8.1%) out of the 531 individuals harbored *P. ovale species *infection”**

**General remark:** *P. ovale curtisi* and *P. ovale wallikeri *are considered two distinct species and not subspecies (see articles by Colin Sutherland for more information on the subject). Please chage subspecies to species in the entire document. **We appreciate the reviewer pointing this out. We have now corrected all the sections in the manuscript where the species was referred to as *P. ovale curtisi*. The sections now read *P. ovale* species.** **Introduction:**

 You cite the work of Miller et al but do not give the prevalence of the *P.ovale *spp they found with their newly developed qPCR. This is interesting information. **In order to detect all *P. ovale* species simultaneously, Miller et al. developed an inclusive P. ovale-specific real-time PCR assay based on conserved regions between *P. ovale curtisi and P. ovale wallikeri* in the reticulocyte binding protein 2 (rbp2) gene. However, their study focused on validation of their new assay using microscopy positive *P. ovale* samples (22 samples) only and not on determining the prevalence of *P. ovale species* in the area. **  

**Results:** You said you used a Ct threshold of 40 for qPCR results, but in the extended Table 3, you gave us positive qPCR results with Ct>40: **All qPCR results associated with all these analysed samples had been previously indicated in our lab book as neg (for all samples with CT>40 or undetermined values) and actual CT values for those with CT values <40. These results are recorded in the main dataset (Akinyi et al Ovale_27June22_de-identified.xlxs). To avoid confusion to the readers, we have opted to remove extended table 3 and only include the main data set which contains complete information on all samples.**

 You wrote “three of eight samples that tested positive by microscopy also tested positive by PCR”-> The remaining five samples were PCR negative? Also for other Plasmodium species? **Yes, the remaining 5 samples were PCR negative for *P. ovale* species. Microscopic examination of these samples for *P. falciparum* and *P. malariae *were both negative. *P. falciparum* PCR data was only available for the primary health facility samples. See below table on result for other species.  We report this under the section on** **sensitivity of microscopy and PCR diagnostic assays in detecting *P. ovale* mono and mixed infections.** **This information is also available on our main data set (Akinyi et al Ovale_27June22_de-identified.xlxs).** **Sample ID** **P. ovale microscopy** **P. ovale PCR** **P falciparum microscopy** **P. malariae microscopy** **P. falciparum PCR**

 P_222 positive negative negative negative positive J_002 positive negative negative negative Not done J_003 positive negative negative negative Not done J_007 positive negative negative negative Not done J_024 positive negative negative negative Not done   In extended table 3, you had 21 isolates with a Ct<40 for P. ovale. But in general able with the combined results of qPCR and conventional PCR you have 31 Isolates with a Ct<40 how do you explain the difference between the two tables? **Extended table 3 was a subset of the main dataset and was included to highlight the expected raw output of the qPCR results. All qPCR results associated with all these analysed samples had been previously indicated in our lab book as neg (for all samples with CT>40) and actual CT values for those with CT values <40. These results are in the main data set (Akinyi et al Ovale_27June22_de-identified.xlxs). To avoid confusion to the readers, we have now opted to only include the main data set which contains complete information on all samples.**

 You said you detected P.ovale curtisi in 48 isolates, but when I looked at the results in the overall table only 43 isolates have a yes in the P.ovale_PCR overall column. Furthermore as only 21 isolates were positive in conventional PCR, how can you differentiate *P.ovale curtisi* from P.ovale wallikeri with q PCR results alone? **Thank you for pointing this out. We have corrected the prevalence to the 43 isolates and have omitted the subspecies names. We now address the isolates as *P. ovale* species.**

 I am convinced that by clearly explaining in the text number the positives by conventional PCR and.or qPCR, and the number of positives to be consistent with the extended table the ariticle will gain in comprehension. **We appreciate the reviewer’s comments and have addressed all the comments.**

# Reviewer report: Reviewer response for version 2

**DOI:** 10.21956/wellcomeopenres.22212.r67418

**Published:** 2023-09-27

**Authors:** Benoit Malleret

**Affiliations:** 1 Department of Microbiology and Immunology, Immunology Translational Research Programme, Yong Loo Lin School of Medicine, National University of Singapore, Singapore, Singapore

No further comments.

Is the work clearly and accurately presented and does it cite the current literature?

Yes

If applicable, is the statistical analysis and its interpretation appropriate?

Not applicable

Are all the source data underlying the results available to ensure full reproducibility?

Yes

Is the study design appropriate and is the work technically sound?

Yes

Are the conclusions drawn adequately supported by the results?

Yes

Are sufficient details of methods and analysis provided to allow replication by others?

Yes

Reviewer Expertise:

Malaria,  Parasitology, Innate Immunity, Microbiome

I confirm that I have read this submission and believe that I have an appropriate level of expertise to confirm that it is of an acceptable scientific standard.

# Reviewer report: Reviewer response for version 1

**DOI:** 10.21956/wellcomeopenres.19922.r60627

**Published:** 2023-06-27

**Authors:** Valentin Joste

**Affiliations:** 1 National Malaria Reference Centre, AP-HP, Hôpital Bichat - Claude Bernard, University of Paris, Paris, France

This study, focusing on *P. ovale curtisi*, is interesting and well-written. The objectives are clear and well-presented. The introduction and discussion are clear. Such studies are needed to better understand the prevalence of the two species of *P. ovale* spp.

 However, this article suffers from a lack of clarity in method that makes it more difficult to read. In particular, I have difficulty understanding how the samples were included and what technique was used for each.

**Method**

1. You have only included samples with positive non-Pf microscopy and have therefore not included negative samples. I assume this is because they were not preserved? I don't understand why you didn't also include samples with positive microscopy for *P. falciparum*. Co-infection with *P. ovale* spp., as you rightly point out, is frequent.
2. I didn't understand how the samples that were tested with PCR or qPCR were chosen.
3. Does qPCR amplify only *P. ovale curtisi*? Or also *P. ovale wallikeri*?
4. Do you plan to test samples with rOVA1v/rOVA2v for *P. ovale wallikeri*?

**Results**

 In my opinion, a flow-chart clearly indicating the number of samples included in each site/tested by PCR/tested by qPCR is absolutely necessary to help readers understand. To illustrate my difficulties, in extended Table 4, if I understand correctly, sample K_054 was positive for *P. ovale* spp. with qPCR (27,41), but I did not find this isolate in extended Table 3. 22 samples had Ct < 40 in extended table 3 while 31 had Ct < 40 in extended table 4.

1. There is a problem in the percentages of females and males that do not reach 100%.
2. I think you should add the % of each species calculated on the total number of samples (as you did) but also on the number of positive samples.
3. " *Three out of the eight samples that tested positive by microscopy also tested positive by PCR*" means that 5 of the 8 samples were negative? Did you have an explanation? Error in the species diagnosing species in microscopy?
4. Please explain how you calculated the sensitivity of the microscopy. What was your gold-standard? The combined results of the conventional PCR and qPCR?
5. As I understand it, the results of conventional PCR and qPCR have been grouped together. I understand that conventional PCR also amplified *P. ovale curtisi*, but you didn't specify whether qPCR also amplified *P. ovale wallikeri*? This would alter the results.
6. For greater clarity, I think a table with PCR and qPCR results would help to understand the results.
7. You write that the primary health care facility had PCR results for *P. falciparum*. You didn't specify this in the method section.

**Discussion**

1. You have reported a higher prevalence of *P. ovale* spp. positive microscopy in the community compared to the primary or tertiary institution. I think you have reversed the terms " *asymptomatic*" and " *symptomatic*" in the paragraph beginning " *In this area of moderate to high transmission*". Indeed, Faye reported a higher prevalence of positive *P. ovale* microscopy in symptomatic patients than in asymptomatic patients.

**General remarks**

1. I recommend to change *P. ovale* with *P. ovale* spp.
2. *P. ovale curtisi* and *P. ovale wallikeri* are more often defined as species rather than subspecies in the literature. Despite the absence of in vitro data validating this postulate, the work of Oguike *et al* provides evidence of non-recombination between the two *P. ovale* species ( http://dx.doi.org/10.1016/j.ijpara.2015.02.004). I recommend that you replace subspecies with species.

Is the work clearly and accurately presented and does it cite the current literature?

Yes

If applicable, is the statistical analysis and its interpretation appropriate?

Partly

Are all the source data underlying the results available to ensure full reproducibility?

Yes

Is the study design appropriate and is the work technically sound?

Partly

Are the conclusions drawn adequately supported by the results?

Yes

Are sufficient details of methods and analysis provided to allow replication by others?

Partly

Reviewer Expertise:

Plasmodium diagnosis; molecular biology

I confirm that I have read this submission and believe that I have an appropriate level of expertise to confirm that it is of an acceptable scientific standard, however I have significant reservations, as outlined above.

## References

[1] : Dimorphism in genes encoding sexual-stage proteins of Plasmodium ovale curtisi and Plasmodium ovale wallikeri. *Int J Parasitol* .2015;45(7) : 10.1016/j.ijpara.2015.02.004 449-54 10.1016/j.ijpara.2015.02.004 25817462 PMC4428623

# Response

**Published:** 2023-09-11

**Authors:** Mercy Akinyi

**Affiliations:** KEMRI Wellcome Trust, Kenya

This study, focusing on P. ovale curtisi is interesting and well written. The objectives are clear and well-presented. The introduction and discussion are clear. Such studies are needed to better understand the prevalence of the two species of *P.ovale spp*.****

**Thank you for the comments.**  

 However, this article suffers from a lack of clarity in method that makes it more difficult to rea. In particular, I have difficulty understanding how the samples were included and what technique was used for each.

**We have addressed this concern by providing details in the methods section responses below.**

 Methods

1. You have only included samples with positive non-Pf microscopy and have therefore not included negative samples. I assume this is because they were not preserved? I don't understand why you didn't also include samples with positive microscopy for *P. falciparum*. Co-infection with *P. ovale* spp., as you rightly point out, is frequent.

**This study was conducted in two phases. The first phase focused on 1) optimizing molecular conventional PCR screening for *P. ovale *and 2) screening community samples *. *During this phase of the study, we first selected non-pf microscopy samples that were available from the community (20 samples) from 2009-2015 to confirm the presence of *P. ovale* in the study area. Upon positive detection and confirmation of the *P. ovale* by conventional PCR screening and sequencing in the first set of samples, we then run all the available 119 community samples (Extended data ovale 4). The second phase of the study comprised of all available archived samples from the tertiary and primary health facilities regardless of their species microscopy positivity status (392 samples). This information has now been included in the methodology section.**

1. How were samples were tested with PCR or qPCR were chosen.

**As mentioned in response 1 above, we had multiple phases of screening.** **The first phase focused on 1) optimizing molecular conventional PCR screening for *P. ovale *and 2) screening community samples** **. During the second phase of the study, we also optimized RT-PCR because it increases the sensitivity to detect more positive samples. We screened** **all available archived samples from the tertiary and primary health facilities regardless of their species microscopy positivity status (392 samples).** **S** **creening** **during this phase** **involved conventional and/or RT-PCR screening and was dependent on available reagents at the time of screening** **.** **However, all** **RT-** **PCR positive samples (except for 3 samples that were accidentally omitted) underwent conventional PCR to enable sanger** **sequencing for species identification.** **We have now provided a flow chart (extended data ovale 4) that clearly indicates which samples went through which test.**
** **  

1. Does qPCR amplify only *P. ovale curtisi*? Or also *P. ovale wallikeri*?

**We agree with the reviewer that the qPCR primers are specific to *P. ovale *species which means they are likely to amplify both species of *P.ovale. *However, all the samples we sequenced were P *ovale curtisi*. **

1. Do you plan to test samples with rOVA1v/rOVA2v for *P. ovale wallikeri*?

**The qPCR primers are specific to *P. ovale *species which means they are likely to amplify both species of *P.ovale. *We were very eager to test the samples for *P.ovale wallikeri* as well. However, we were unable to secure a reliable positive control for the same at the time. We have however received funding to extend this study with a bigger sample set and we anticipate testing for this subspecies as well.** **Results**

 1. In my opinion, a flow-chart clearly indicating the number of samples included in each site/tested by PCR/tested by qPCR is absolutely necessary to help readers understand.

**We welcome this suggestion by the reviewer. We acknowledge the utility of assisting the readers understand the sample sets available and their associated tests. We have now added a flow chart depicting the same (Extended data ovale 4)**  

 2. To illustrate my difficulties, in extended Table 4, if I understand correctly, sample K_054 was positive for *P. ovale* spp. with qPCR (27,41), but I did not find this isolate in extended Table 3. 22 samples had Ct < 40 in extended table 3 while 31 had Ct < 40 in extended table 4.  

**We apologize for this. We recorded our RT-PCR results in our master project sheets with CT values if positive and ‘neg’ if negative (This is available in our main de-identified data set). However, the journal when requested to provide raw RT-PCR to support our work, we were only able to retrieve a subset of the raw data from the RT-PCR machine (extended data table 3). This explains the discrepancies between the extended tables 3 and the main data set (previously described as extended table 4 by the reviewer).**

 3.There is a problem in the percentages of females and males that do not reach 100%.

**Thank you for pointing this out. We have now corrected the percentage for males to read 49.9% instead of 40.9%.**

****4.I think you should add the % of each species calculated on the total number of samples (as you did) but also on the number of positive samples.

**We thank the reviewer for this suggestion. We have added a column containing this information on table 1**.

 5." *Three out of the eight samples that tested positive by microscopy also tested positive by PCR*" means that 5 of the 8 samples were negative? Did you have an explanation? Error in the species diagnosing species in microscopy?

**Second, we had discussed this error in the discussion section. *“It is evident that there is an underestimation of P. ovale prevalence in malaria endemic countries because overall microscopy identified about five-fold fewer infections compared to PCR of the highly conserved 18sRNA gene. The low sensitivity of microscopic diagnosis can be attributed to low parasite densities or lack of skilled microscopists with sufficient experience to morphologically differentiate P.ovale from other malaria species.”***

 6.Please explain how you calculated the sensitivity of the microscopy. What was your gold-standard? The combined results of the conventional PCR and qPCR

**We have now added a description on how we calculated sensitivity and specificity. We used the combined results of the conventional and qPCR as our gold standard. We first generated a 2 by 2 matrix table (named “dat”) as shown below then used the epiR package in R statistical software to conduct the specificity and sensitivity tests. Please see code below.** **PCR+    PCR-** **Microscopy+    3             5** **Microscopy-   40          483** **install.packages(“epiR”)** **library(epiR)** **rval <- epi.tests(dat, conf.level = 0.95)** **print(rval); summary(rval)**

1. As I understand it, the results of conventional PCR and qPCR have been grouped together. I understand that conventional PCR also amplified *P. ovale curtisi*, but you didn't specify whether qPCR also amplified *P. ovale wallikeri*? This would alter the results.

**We agree with the reviewer that the qPCR primers are specific to *P. ovale *species which means they are likely to amplify both species of *P.ovale. *This could possibly explain why some of the RT-PCR were negative upon amplification with *P.ovale* curtisi primers. We have included this statement in the discussion. As we expand our study, we will take this into consideration.**

1. For greater clarity, I think a table with PCR and qPCR results would help to understand the results.

**We have included this information in our extended data ovale 4, table 2. **

1. The health care facility had PCR results for *P. falciparum*. You didn't specify this in the method section.

**We retrieved these results from a previously concluded project. We have now added the same in the methods.** **In addition, we retrieved *P. falciparum* PCR screening results for the tertiary healthcare facility to aid in identification of co-infections. These results generated from a previous study that had just concluded.** **Discussion**

1. You have reported a higher prevalence of *P. ovale* spp. positive microscopy in the community compared to the primary or tertiary institution. I think you have reversed the terms " *asymptomatic*" and " *symptomatic*" in the paragraph beginning " *In this area of moderate to high transmission*". Indeed, Faye reported a higher prevalence of positive *P. ovale* microscopy in symptomatic patients than in asymptomatic patients.

**Thank you for this correction. We have now amended it.** **General remarks**

1. I recommend to change *P. ovale* with *P. ovale* spp.

**Thank you for this comment. We have now revised our title to state *P.ovale* species**

1. *P. ovale curtisi* and *P. ovale wallikeri* are more often defined as species rather than subspecies in the literature. Despite the absence of in vitro data validating this postulate, the work of Oguike *et al* provides evidence of non-recombination between the two *P. ovale* species ( http://dx.doi.org/10.1016/j.ijpara.2015.02.004). I recommend that you replace subspecies with species.

**Thank you for this comment. We have now revised our title to state *P.ovale* species**

# Reviewer report: Reviewer response for version 1

**DOI:** 10.21956/wellcomeopenres.19922.r51982

**Published:** 2022-08-31

**Authors:** Benoit Malleret

**Affiliations:** 1 Department of Microbiology and Immunology, Immunology Translational Research Programme, Yong Loo Lin School of Medicine, National University of Singapore, Singapore, Singapore

The manuscript "A cross-sectional analysis identifies a low prevalence of *Plasmodium ovale curtisi* infections in symptomatic and asymptomatic individuals in Kilifi county, Kenya.” by Akinyi et al. describes the proportion of *Plasmodium falciparum*, *P. malariae* and *P. ovale* positive cases detected by microscopy and PCR in 531 samples . The samples were collected in Kilifi county between 2009 and 2020. I do not have any concerns regarding the methodology and the article is well-written.

 The authors need to specify that the low parasitemia of *Plasmodium ovale* is associated to the cell tropism of these parasites, that invade preferentially immature red blood cells (reticulocytes). Like *Plasmodium vivax*, the parasites for *P. ovale* could be mainly in the bone marrow so the detection of the parasitemia in the peripheral blood represent only the peak of the iceberg.

 A point of discussion is missing concerning the phylogenetic tree (Figure 2).

 Minor points:

 Page 3: Reticulocyte binding protein 2 gene abbreviation is rbp2 and not rb2.

 For further studies, the authors should include *Plasmodium vivax* primers for the PCR detection.

Is the work clearly and accurately presented and does it cite the current literature?

Yes

If applicable, is the statistical analysis and its interpretation appropriate?

Not applicable

Are all the source data underlying the results available to ensure full reproducibility?

Yes

Is the study design appropriate and is the work technically sound?

Yes

Are the conclusions drawn adequately supported by the results?

Yes

Are sufficient details of methods and analysis provided to allow replication by others?

Yes

Reviewer Expertise:

Malaria,  Parasitology, Innate Immunity, Microbiome

I confirm that I have read this submission and believe that I have an appropriate level of expertise to confirm that it is of an acceptable scientific standard, however I have significant reservations, as outlined above.

# Response

**Published:** 2023-09-11

**Authors:** Mercy Akinyi

**Affiliations:** KEMRI Wellcome Trust, Kenya

The manuscript "A cross-sectional analysis identifies a low prevalence of *Plasmodium ovale curtisi* infections in symptomatic and asymptomatic individuals in Kilifi county, Kenya.” by Akinyi et al. describes the proportion of *Plasmodium falciparum*, *P. malariae* and *P. ovale* positive cases detected by microscopy and PCR in 531 samples. The samples were collected in Kilifi county between 2009 and 2020. I do not have any concerns regarding the methodology and the article is well-written.

**Thank you for these comments.**  

 The authors need to specify that the low parasitemia of *Plasmodium ovale* is associated to the cell tropism of these parasites, that invade preferentially immature red blood cells (reticulocytes). Like *Plasmodium vivax*, the parasites for *P. ovale* could be mainly in the bone marrow so the detection of the parasitemia in the peripheral blood represent only the peak of the iceberg.

**We welcome this suggestion and have now included it in the discussion. *“*** ***Low parasitemia (often below 5%) in P. ovale infected individuals is not uncommon because these parasites only infect young erythrocytes (reticulocytes) (Okafor and Finnigan, 2023)”.***

 A point of discussion is missing concerning the phylogenetic tree (Figure 2)

**We discuss the phylogenetic tree under the results section. We have now added a few sentences in the discussion to reflect the same “**. ***Phylogenetic analysis showed that the sequences retrieved from this study was within a well-supported P. ovale spp. Clade. The close homology (99% to 100% identity) of our sequences with the West African and China-Mynamar P. ovale curtisi isolates confirms that the*** ***18S rRNA*** ***locus is highly conserved in this species and hence is an accurate diagnostic tool that should continue to be used for detection and identification of this species globally*** ***”***

**Minor points**

 Page 3: Reticulocyte binding protein 2 gene abbreviation is rbp2 and not rbs****

**Thank you for the correction. We have edited the abbreviation. **  

 For further studies, the authors should include *Plasmodium vivax* primers for the PCR detection.

**Screening for *P.vivax* was limited for this preliminary study. However, we are considering its inclusion in a current non-falciparum screening study in western and coastal Kenya.**
